# Rowdyism *versus* Education

**Published:** 1903-09-19

**Authors:** 


### Editor's Letter-Box.

**[Our Correspondents are reminded that prolixity is a great bar to publication, and that brevity of style and conciseness of statement greatly facilitate early insertion.]**

### ROWDYISM *VERSUS* EDUCATION.

Mr. S. Edward Pedley writes: Your remarks on the Passive Resistance movement against the present Educational Act appearing under Annotations in your issue of August 22nd are entirely out of place in a paper supposed to be free from sectarian bias and devoted to medical science and hospital administration. The statements as to rowdy preachers and baser sort of hired politicians are grossly insulting, aimed as they are at the honoured leaders of the Free Churches and many lovers of religious liberty. Not a few among the Passive Resisters belong to the medical profession, men honoured for their ability as well as moral courage. The present Government is well designated a Khaki Government. Its leaders appealed for votes at the General Election to approve and carry on to completion the Boer War, not to carry out a revolutionary policy of putting Rome and Anglicanism on the rates, nor for the proselytising of the children of Free Churchmen under the plea of education.

I am confident that many of your readers, like myself, consider your comments on this great national protest—not "organised rowdyism"—against denominational education as unfair, ungenerous, and untrue. I am aware that this letter will not be published, though it affords me some satisfaction as one of your readers to enter my protest against your scandalous remarks.

